# Effect of Natural Antioxidants from Fruit Leaves on the Oxidative Stability of Soybean Oil during Accelerated Storage

**DOI:** 10.3390/antiox11091691

**Published:** 2022-08-29

**Authors:** Hanem M. M. Mansour, Sobhy Ahmed El-Sohaimy, Ahmed M. Zeitoun, Eman M. Abdo

**Affiliations:** 1 Department of Food Technology, Arid Lands Cultivation Research Institute (ALCRI), City of Scientific Research and Technological Applications (SRTA-City), New Borg El Arab, Alexandria P.O. Box 21934, Egypt or; 2 Department of Technology and Organization of Public Catering, Institute of Sport, Tourism and Service, South Ural State University (SUSU), 454080 Chelyabinsk, Russia; 3 Department of Food Science, Faculty of Agriculture (Saba Basha), Alexandria University, Alexandria P.O. Box 21531, Egypt

**Keywords:** oxidative degradation, rancidity, pomegranate leaves, guava leaves, grape leaves, bioactive components, plant wastes

## Abstract

Plant by-products are safe, sustainable, and abundant natural antioxidant sources. Here we investigated the antioxidant activity of a mixture of lyophilized pomegranate, guava, and grape (PGG) leaves water extract (1:1:1) and examined its ability to retard the rancidity of soybean oil during accelerated storage at 65 °C for 30 days. To achieve this, we evaluated the oxidative stability of soybean oil enriched with PGG extract at 200, 400, and 800 ppm. We also compared the effect of PGG extract with butylated hydroxytoluene (BHT) (400/100 ppm) with that of only BHT (200 ppm). We observed that 8.19 and 1.78 µg/mL of the extract could scavenge 50% of DPPH^•^ and ABTS^•^, respectively, indicating its enhanced antioxidant activity. Enriching soyabean oil with the extract at 800 ppm improved its oxidative stability by reducing the acid value to 1.71 mg/g and the total oxidation to 99.87 compared to 2.27 mg/g and 150.32 in the raw oil, respectively. Moreover, PGG-800 ppm inhibited oxidation by 46.07%. Similarly, PGG-400 ppm reinforced BHT (100 ppm) to provide oxidative stability as BHT (*p* > 0.05), with TOTOX values of 87.93 and 79.23, respectively. PGG-800 ppm and PGG/BHT mix potently inhibited the transformation of polyunsaturated fatty acids into saturated ones. Therefore, the PGG extract might be an efficient substitute for BHT (partially or totally) during industrial processes.

## 1. Introduction

Vegetable oil, including corn, sunflower, and soybean, are commonly used in food preparation [1]. Soybean oil is the second most frequently used oil after palm oil, with annual consumption and production of 59.48 [2] and 60.27 million tons, respectively [3]. It contains high concentrations of polyunsaturated fatty acids, mainly linoleic [4] and linolenic acids [1], which leads to rapid deterioration. The speed of the rancidity process depends on many factors, such as storage.

Improper storage of vegetable oils—exposing them to light, high temperature, oxygen, and moisture—triggers the rancidity process [4] via various reactions, including hydrolysis, oxidation, polymerization, and isomerization [5]. Once the rancidity occurs, several undesirable compounds are formed that impart bad odor and flavor and shorten the shelf-life of these oils [1,4,6]. It also negatively affects the oil quality, nutritional value, and acceptability [7], resulting in economic losses [6]. When consumed, it causes non-communicable diseases, including cardiovascular diseases, cancer, and aging [4,6]. Besides, the free radicals in rancid oil are mutagenic [8]. Hence, antioxidants are essential to retard the oxidation process and extend the shelf-life of these oils [9].

Synthetic antioxidants, including butylated hydroxyanisole (BHA), butylated hydroxytoluene (BHT), tert-butylhydroquinone (TBHQ), and propyl gallate (PG), are widely used in the oil industry to retard oxidative rancidity [6,10]. According to the generally recognized safe list, the maximum concentration for BHA and BHT is 200 ppm. At the same time, for TBHQ and PG, it is less than 200 ppm [11] to prolong the oil’s oxidative stability. However, synthetic antioxidants are unstable at high temperatures and can cause toxicity in short-term and long-term use when stored for prolonged periods [12]. Besides, consumers prefer natural antioxidants over synthetic ones [13]. Hence, exploring safe, sustainable, and cheap natural antioxidant sources is crucial.

Agro-wastes are sustainable, safe, low-cost, and rich sources of natural antioxidants [1,4,10]. Studies proved that plant by-product (peels, pomaces, industrial by-products, and essential oils from plant wastes) could enhance the oxidative stability of vegetable oils. Pomegranate peel extract was more efficient than TBHQ in inhibiting soybean, corn, and sunflower oil oxidation [1]. Mangosteen peel extracts retarded lipid peroxidation of sunflower oil [6]. In addition to retarding oxidative rancidity, essential oils extracted from plant by-products also impart flavor to these oils. For example, the essential oils extracted from *Punica granatum cv. Heyinshiliu* peels enhanced the stability of sunflower oil during storage for up to 12 months [7]. Although plant leaves are potent by-products, their effect on retarding oil oxidation remains unexplored [14,15]. Besides, to our knowledge, no previous studies investigated the effect of incorporating a mixture of natural antioxidants in the oil industry.

Pomegranate (*Punica granatum* L.) [16,17,18], guava (*Psidium guajava* L.) [19,20], and grape (*Vitis vinifera* L.) leaves [21,22] are safe and rich sources of natural antioxidants and pigments but are underutilized. Here, we aimed to explore their potential as novel sustainable alternatives to synthetic antioxidants. Therefore, we investigated the effect of enriching soybean oil with a mixture of pomegranate-guava-grape leaf extracts (1:1:1) (PGG) on retarding the rancidity compared to the synthetic antioxidant BHT. We also studied the synergistic effect of the PGG extract on BHT at concentrations < 200 ppm in preventing oil deterioration during accelerated storage at 65 °C for 30 days.

## 2. Materials and Methods

### 2.1. Materials

Pomegranate (*Punica granatum* L.), guava (*Psidium guajava* L.), grape (*Vitis vinifera* L.) leaves, and frozen French fries were procured from a local market in Alexandria, Egypt. The refined soybean oil (without antioxidants) was commercially obtained from Borg Al Arab, Alexandria, Egypt.

2,2, diphenyl-1-picrylhydrazyl (DPPH), 2,2′-azino-bis (3-ethylbenzothiazoline-6-sulfonate) (ABTS), Folin-Ciocalteu, Thiobarbituric acid (TBA), *p*-anisidine, Butylated hydroxytoluene (BHT), and phenolic standards are purchased from Merck, Darmstadt, Germany. Isooctane was collected from Loba Chemie Pvt Ltd., Mumbai, Maharashtra, India. Chloroform, ethanol, glacial acetic acid, potassium iodide, and starch are obtained from Aljumhoria company for chemicals, Alexandria, Egypt.

### 2.2. Extraction of Bioactive Components

After carefully washing the three types of leaves under running water, they were dried at 50 ± 2 °C for 24 h in an oven (Wt-binder, Bohemia, NY, USA). The dried leaves were ground, and the powders were separately mixed with boiled water at 100 °C (1:50 w:v) for 10 min. Afterward, the mixture was stirred for 3 h at room temperature and centrifugated at 3000 rpm for 10 min at 20 °C. The supernatant was filtered and lyophilized in a vacuum freeze dryer (FDE 0350, Humanlab Inc., Bucheon-si, Gyeonggi-do, Korea) [23]. The lyophilized extracts and combined pomegranate/guava/grape (PGG) leaf extracts (1:1:1) were stored at −18 ± 2 °C for future use as oil additives.

### 2.3. Determination of Bioactive Components

To estimate the phenolics, flavonoids, and antioxidant activity, we liquefied 1 mg of each lyophilized leaf extract and the PGG mixture in 5 mL d.H_2_O [1].

#### 2.3.1. Total Phenolics

The total phenolic content was determined as described by Vodnar et al. [24]. One ml Folin-Ciocalteu reagent (0.2N) and 800 µL sodium carbonate (7.5%) were added to 200 µL of each extract in a tube. After incubating the mixture for 2 h in the dark, its absorbance was measured at 760 nm using a spectrophotometer (Jenway 6405UV/VIS, Stone, Staffordshire, UK). The total phenolic content was expressed as mg/g of gallic acid.

#### 2.3.2. Total Flavonoids

To estimate the flavonoid content, we incubated a mixture of 1 mL of the extract, 4 mL of d.H_2_O, and 300 µL of sodium nitrite (5%) for 5 min before adding 300 µL of aluminum chloride (10%) and re-incubating the mixture for another 6 min. Then, 2 mL of NaOH (1 mol/mL) was added, and the mixture volume was made up to 10 mL with d.H_2_O. Then, the absorbance was measured at 510 nm, and the flavonoid content was expressed as mg/g catechin [25].

### 2.4. Total Phenolics Profile

We used an HPLC system (Agilent, Agilent 1260 series, Stevens Creek BLVD, San Jose, CA, USA) with an Eclipse C18 column (4.6 mm × 250 mm, 5 μm) and a multi-wavelength detector to measure the phenolics in the PGG water extract at 280 nm. The PGG extract (5 μL) was injected into the column. The mobile phase consisted of two solvents: Solvent A (water) and solvent B (99.95 acetonitrile: 0.05 trifluoroacetic acid). The elution was performed at 40 °C by a linear gradient flow rate of 1 mL/min for 20 min as follows: 0 min (82% A); 0–5 min (80% A); 5–8 min (60% A); 8–12 min (60% A); 12–15 min (82% A); 15–16 min (82% A); 16–20 min (82% A).

### 2.5. Antioxidant Activity

#### 2.5.1. DPPH^•^ Free Radical Scavenging Assay

The ability of the extract to scavenge 1,1-diphenyl-2-picrylhydrazyl (DPPH^•^), a free radical, was determined by mixing 0.5 mL of the extract with 0.5 mL of a freshly prepared methanolic DPPH^•^ (0.3 mM) and incubating the mixture for 20 min at room temperature. Afterward, the absorbance values of the mixture and control (DPPH^•^ solution) were measured at 517 nm [26]. The inhibition ratio % was calculated according to Equation (1) as follows:

(1) $$\%\;\mathrm{Inhibition}=\frac{\mathrm{absorbance}\;\mathrm{of}\;\mathrm{control}\;-\;\mathrm{absorbance}\;\mathrm{of}\;\mathrm{sample}}{\mathrm{absorbance}\;\mathrm{of}\;\mathrm{control}}\times 100$$

#### 2.5.2. ABTS^•^ Radical Scavenging Activity

ABTS^•^ (2,2′-azino-bis(3-ethylbenzothiazoline-6-sulfonic acid)) solution (7 mmol/L) and potassium persulfate (2.4 mmol/L) were mixed in equal volumes and incubated in the dark for 16 h. The mixture was diluted with d.H_2_O (1:60 v:v) to prepare a reagent with an absorbance of 0.701 ± 0.01 at 734 nm. The reagent (4 mL) was mixed with the extract (10 µL) and incubated for 6 min before measuring the absorbance at 734 nm versus the control [27]. The ABTS^•^ radical scavenging activity was calculated using Equation (2) as follows:

(2) $$\mathrm{ABTS}\;\mathrm{radical}\;\mathrm{scavenging}\;\%=\frac{\mathrm{absorbance}\;\mathrm{of}\;\mathrm{control}\;-\;\mathrm{absorbance}\;\mathrm{of}\;\mathrm{sample}}{\mathrm{absorbance}\;\mathrm{of}\;\mathrm{control}}\times 100$$

### 2.6. Oil Preparation

The oil samples were prepared using the PGG mixture (1:1:1) and the synthetic antioxidant (BHT) (Table 1). The extract was dissolved in preheated oil (50 °C) as described by Chong et al. [6].

### 2.7. Storage Conditions

The raw and enriched (with different concentrations of PGG) soybean oil samples, BHT, and PGG/BHT (1200 mL each) were stored at 65 °C for 30 days. According to Chong et al. [6], a day of storage at 65 °C equals a month of storage at ambient temperature. Accordingly, we collected 200 mL of each sample every six days to determine the oil’s oxidative stability. The fatty acids composition was simultaneously analyzed after 0, 6, and 30 days of storage.

### 2.8. Determination of Physicochemical and Oxidative Stability of the Oil

#### 2.8.1. Acid Value (AV)

Petroleum ether (50 mL) and phenolphthalein were mixed with 10 g of the oil sample before titration with 0.1 M of potassium hydroxide, which was continued until the pink color developed [28]. The AV was calculated using Equation (3) and expressed in (mg KOH/g oil).

(3) $$\mathrm{Acid}\;\mathrm{value}\;\left(\mathrm{mg}/g\right)=\frac{\left(V-\mathrm{Vb}\right)\times N\times 56.1}{W}$$

where (V) and (Vb) are the volumes of potassium hydroxide used for titrating the sample and the blank, respectively; (N) is the potassium hydroxide normality; and (W) is the weight of the sample.

#### 2.8.2. Peroxide Value (PV)

In a flask, the oil sample (5 g) with 50 mL of acetic acid/chloroform (3:2 v:v) and saturated potassium iodide (1 mL) were shaken for five minutes in the dark before adding 100 mL of d.H_2_O. The mixture was titrated with sodium thiosulfate (0.01 N) until the yellow color disappeared. Then, 5 mL of starch indicator (1%) was added to the mixture and shaken vigorously during re-titration until the blue color faded [29]. The PV was calculated using Equation (4) and expressed in (meq O_2_/kg).

(4) $$\mathrm{Peroxide}\;\mathrm{value}\;\left({\mathrm{meqO}}_{2}/\mathrm{kg}\right)=\frac{\left(V-\mathrm{Vb}\right)\times N\times 1000}{W}\;$$

where (V) and (Vb) are the volumes of sodium thiosulfate used for titrating the sample and the blank, respectively; (N) is the normality of sodium thiosulfate; and (W) is the weight of the oil sample.

#### 2.8.3. Inhibition of Oxidation (IO%)

IO% of the oil samples was calculated as illustrated by Metzner Ungureanu et al. [30] using the following Equation (5):

(5) $$\mathrm{IO}\%\;=1-\frac{\mathrm{the}\;\mathrm{increase}\;\mathrm{in}\;\mathrm{the}\;\mathrm{PV}\;\mathrm{of}\;\mathrm{oil}\;\mathrm{sample}}{\mathrm{the}\;\mathrm{increase}\;\mathrm{in}\;\mathrm{the}\;\mathrm{PV}\;\mathrm{of}\;\mathrm{control}}\times 100$$

#### 2.8.4. *p*-Anisidine Value (*p*-AV)

The absorbance of the oil/iso-octane mixture (2:25 w:v) was measured at 350 nm versus that of iso-octane as a blank (A1). Then, 5 mL of this mixture was combined with *p*-anisidine/glacial acetic acid (0.25:100 *w/v*) for 10 min before measuring the absorbance at 350 nm against the blank containing iso-octane and *p*-anisidine (A2) [30]. *p*-anisidine value (*p*-AV) was calculated using Equation (6) as follows:

(6) $$p-\mathrm{AV}=\frac{25\times \left(1.2\times A2-A1\right)}{W}$$

where (A1) and (A2) are the absorbance values of the oil sample in iso-octane before and after adding *p*-anisidine, respectively, and (W) is the oil sample weight (g).

#### 2.8.5. Total Oxidation Value (TOTOX)

The TOTOX value, which reflects the oxidative degradation status of the oil, was estimated as described by Metzner Ungureanu et al. [30] using Equation (7):

TOTOX = 2 × PV + *p*-AV (7)

where (PV) and (*p*-AV) are the peroxide and para anisidine values, respectively.

#### 2.8.6. Thiobarbituric Acid Reactive Substances (TBARS)

TBARS were determined as described by Drinić et al. [28]. The oil sample (1 mL) was mixed with 3.5 mL thiobarbituric acid (TBA) solution, a mixture of hydrochloric acid (0.25 M), trichloroacetic acid (15%), and thiobarbituric acid (0.375%). The oil/TBA mixture was heated at 100 °C in a water bath till it turned pink. Then, the mix was cooled down under running water and centrifuged at 3000 rpm. The absorbance of the collected supernatant was measured at 532 nm. TBARS values of the oil samples were calculated in mg MDA/kg oil using Equation (8) based on the malondialdehyde (MDA) standard curve equation [4].

(8) $$\mathrm{TBARS}\;(\mathrm{mg}/\mathrm{kg})=\frac{\left(\frac{\mathrm{Abs}-b}{a}\right)\times v\times 72.06}{w\times 1000}$$

where (Abs) is the absorbance of the sample; (a) and (b) are the slope and the intercept from the standard curve; (v) is the final volume of the mixture; and (w) is the sample weight.

#### 2.8.7. Conjugated Dienes (CD) and Trienes (CT)

The filtered oil sample (1 mL) was mixed with 100 mL iso-octane, and its absorbance was measured at 232 nm and 286 nm to determine the CD and CT, respectively [31], using Equation (9):

(9) $$\mathrm{CD}\;\mathrm{and}\;\mathrm{CT}\;\%=\frac{A\;\left(\lambda \right)}{\omega }$$

where (*A*(λ)) is the absorption of the sample at 232 and 270 nm, and (ω) is the concentration of the oil sample (g/100 mL).

#### 2.8.8. Refractive Index (RI)

As described by Neves et al., the RI (^20^ND) of oil samples was measured by a digital refractometer NR101 [5].

#### 2.8.9. GC Analysis of Changes in the Fatty Acids during Accelerated Storage

We prepared the fatty acids methyl ester (FAME) of soybean oil as described by Meng et al. [8]. The oil sample (1 g) was mixed with 12 mL of 2% NaOH-methanol solution, heated at 80 °C, and shaken until the oil completely disappeared. Then, 14 mL of boron trifluoride BF_3_ (15%) was added after cooling the mixture, followed by 30 mL of n-hexane and 100 mL of saturated NaCl. The samples were shaken for 10 min before adding 10 g of anhydrous Na_2_SO_4_ to remove the water. The fatty acid composition from the supernatant was analyzed using gas chromatography (Scion 456-GC, Goes, Stanleyweg 4, The Netherland).

FAME (1 µL) was injected into the gas chromatography equipped with FID (Flame Ionization Detector) and capillary Rt-2560 column (100 m long × 0.25 mm I.D. × 0.25 µm film thickness). The oven temperature was increased from 170 °C to 220 °C with increments of 4 °C/min and then to 250 °C at 1 °C/min. The injector and the detector were at 220 °C and 250 °C, respectively. Helium was used as the carrier gas at a flow rate of 1 mL/min. The formed methyl esters were identified by comparing their retention time to standard methyl esters of fatty acids.

### 2.9. Statistical Analysis

The data were analyzed using two-way ANOVA to determine the effectiveness of the additives with the IBM SPSS program 25, Armonk, New York, United States. The differences between means were compared using one-way ANOVA and Duncan’s test at a confidence level of 95% (*p* < 0.05). The obtained data were expressed in mean ± standard deviation.

## 3. Results and Discussion

### 3.1. Total Phenolics and Flavonoids Content of the Extract

Pomegranate leaves contain approximately eight and six times more phenolics than the grapes and guava leaves (Table 2). Adding pomegranate leaf extract to the grape and guava leaf extracts enhanced the total phenolic content by approximately 3–4 times. Similarly, pomegranate leaf extract had the highest flavonoid content, followed by the guava and grape ones. As expected, the flavonoid content of the PGG mixture was increased by 14.4 and 22.5% compared to the guava and grape leaf extracts, respectively.

### 3.2. Phenolics Profile

The phenolic acids and derivatives in the PGG mixture were more abundant than the flavonoids (Table 3), comprising 96% of the total phenolics in the extract. Ellagic acid was predominant (60%) among the phenolics, followed by chlorogenic acid (22%), gallic acid (9%), and caffeic acid (4%). The high ellagic acid content in the mixture was most likely from the pomegranate leaves, which contained the highest ellagic acid concentration [32] compared to that from guava [33] and grape leaves [34]. Flavonoids accounted for only 4% of the detected phenolics in the PGG extract, with naringenin being the highest flavonoid (2% of the phenolics), followed by rutin (1%).

### 3.3. Antioxidant Activity

As pomegranate leaves had the highest phenolic and flavonoid content, they displayed the highest DPPH^•^ and ABTS^•^ scavenging activity. Adding pomegranate extract, even in small amounts, enabled scavenging of 50% of the DPPH^•^ (11.11 µg/mL) and ABTS^•^ radicals (0.58 µg/mL) compared to guava and grape leaf extracts, indicating its boosting effect on the antioxidant activity of the mixture. Interestingly, 8.19 µg/mL of PGG mixture could scavenge 50% of the DPPH^•^ radicals, indicating a synergistic effect in the PGG mixture’s antioxidant activity compared to each extract separately. Similarly, the PGG extract showed significantly enhanced ABTS^•^ scavenging activity (*p* < 0.05) (Table 2). This might be because mixing extracts with different antioxidant mechanisms reinforce the scavenging activity of the additives [9]. Additionally, the naturally present chlorophyll in the plant leaves [35,36] might also boost the antioxidant activity of the mixture. Moreover, the high antioxidant activity of the extract could be attributed to the high ellagic acid content.

### 3.4. Acid Value (AV)

AV reflects the degree of hydrolysis of the oil [28] and is used to indicate the oil’s rancidity [7]. AV denotes the extent of lipid hydrolysis [10] and hydroperoxide decomposition [5] into free fatty acids and carbonyl groups, respectively. PGG/BHT mixture had a superior anti-hydrolytic effect compared to PGG-800 ppm (*p* < 0.05), and this inhibition was concentration-dependent (*p* < 0.05) compared to the raw oil.

**Figure 1:**
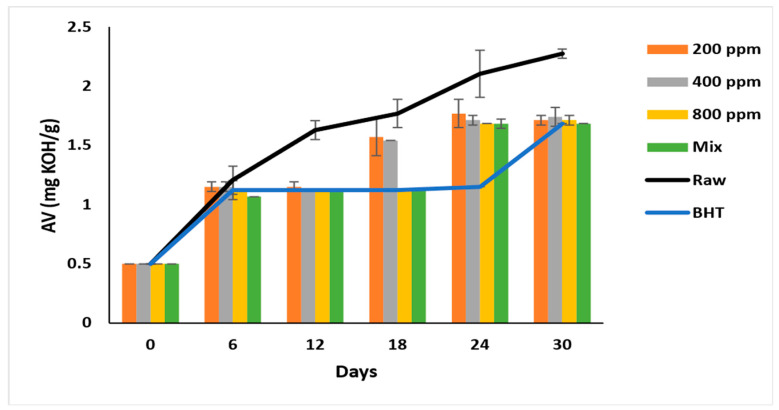
Acid value (mg KOH/g oil) of raw soybean oil (without antioxidants) and soybean oil enriched with PGG leaves extract (200, 400, and 800 ppm), BHT (200 ppm), and a PGG/BHT mixture (400/100 ppm) during the accelerated storage days (30 days) at 65 °C.

The AV of all samples nearly doubled after six days of accelerated storage, ranging between 1.07 ± 0.08 to 1.21 ± 0.12. The formation of free acids gradually increases during storage and reaches 2.27 ± 0.04 mg/g in raw soybean oil (Figure 1). BHT (200 ppm) steadily inhibited the lipid hydrolysis from day 6 till day 24 of the storage, with the AV ranging between 1.12 to 1.15 mg/g, which reached 1.68 ± 0.00 on day 30. PGG-800 ppm was as effective as BHT (*p* > 0.05) in inhibiting lipid hydrolysis after 30 days of accelerated storage. Interestingly, the synergistic effect of PGG (400 ppm) + BHT (100 ppm) was similar to BHT on day 30 of storage. Consequently, PGG-800 ppm and PGG/BHT mix were most efficient (*p* < 0.05) in inhibiting hydrolysis. This result was consistent with Wang et al. [10] and Drinić et al. [28], who reported the synergistic effect of the essential oils from *Angelica dahurica cv. Yubaizhi* (400 ppm) and pomegranate peel extract (500 ppm) on TBHQ and BHT at 100 ppm, respectively. The inhibitory effect of PGG-800 ppm indicates efficient inhibition of lipid hydrolysis similar to that seen in pomegranate peel (1000 ppm) [28] and prickled broom ethanolic (1000 mg/L) extracts [5] on pomegranate and sunflower oils, respectively.

### 3.5. Peroxide Value (PV)

Peroxides and hydroperoxides are the initial oxidation products [7], which are unstable, odorless, and colorless [30]. PV measures oil’s dissolved oxygen [29] and reflects the oil quality [37]. According to Codex Alimentarius [38], the PV of refined oils should not exceed 10 meq O_2_/kg.

PGG/BHT mix followed BHT (*p* < 0.05) in inhibiting peroxide formation. However, PGG-mediated inhibition was concentration-dependent, unlike that in raw oil. Hence, PGG-800 ppm is the best additive compared to other PGG concentrations (*p* < 0.05).

**Figure 2:**
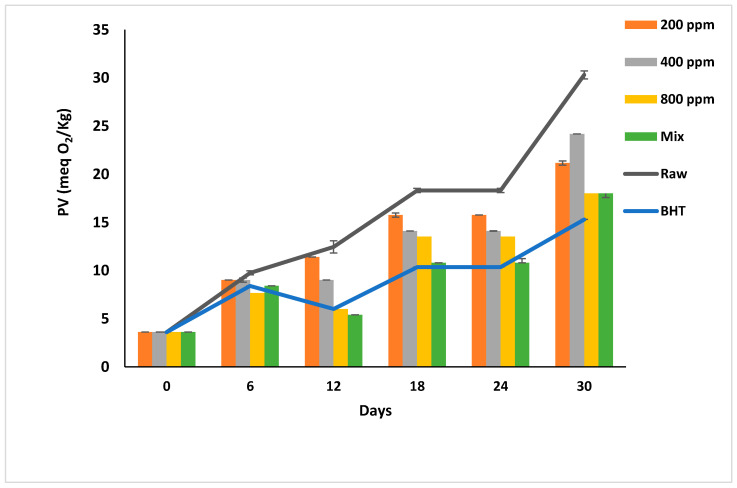
Peroxide value (meq O_2_/kg) of raw soybean oil (without antioxidants) and soybean oil enriched with PGG leaves extract (200, 400, and 800 ppm), BHT (200 ppm), and a PGG/BHT mixture (400/100 ppm) during the accelerated storage days (30 days) at 65 °C.

PGG-400 ppm and PGG-800 ppm were as effective as BHT (*p* > 0.05) in retarding the peroxidation till day 12 of storage. The PV of oil samples was less than the limit (10 meq O_2_/kg) till day 12 of storage compared to PGG-200 ppm and raw soybean oil that turned rancid after day 6 of accelerated storage (Figure 2). Interestingly, the effects of PGG-400 ppm were similar to BHT (100 ppm) as it efficiently inhibited the early stages of oxidation. From day 18 onward, the additives could not inhibit the peroxidation, and the hydroperoxide levels exceeded the limit in all oil samples. However, PGG-800 ppm and PGG/BHT effectively inhibited oil oxidation even after 30 days of accelerated storage to reach a PV of 18 ± 0.00 meq O_2_/kg and 18 ± 0.42 meq O_2_/kg, respectively, compared to 30.3 ± 0.42 meq O_2_/kg of raw soybean oil. The effect of PGG-800 up to 12 days of storage was consistent with the study by El-Hadary and Taha [1], showing that pomegranate peel extract (600 ppm) prevented the rancidity of soybean oil by maintaining the PV below 10 meq O_2_/kg after 10 days of accelerated storage at 70 °C. During the prolonged storage period, our results agreed with Phuong et al. [4], reporting that rambutan peel extract prevents soybean oil oxidation up to 80 days of storage at 30 °C in the dark, and Mohammadi et al. [39]. They showed that enriching soybean oil with nano-encapsulated olive leaves with pectin (300 ppm) reduced the peroxidation of soybean oil during accelerated storage for 20 days—similarly, the essential oils from *Angelica dahurica cv. Yubaizhi* at 400 ppm [10] and pomegranate peel extract at 500 ppm [28] potentiated the anti-peroxidative effect of TBHQ and BHT (100 ppm), respectively.

### 3.6. Inhibition of Oxidation (IO%)

**Figure 3:**
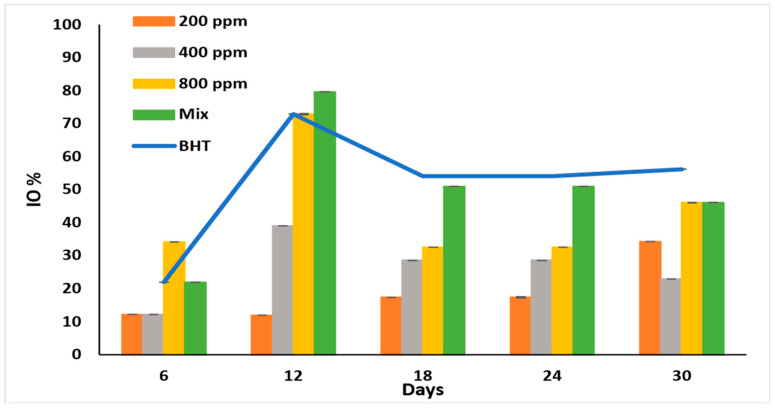
Inhibition of oxidation (IO%) of raw soybean oil (without antioxidants) and soybean oil enriched with PGG leaves extract (200, 400, and 800 ppm), BHT (200 ppm), and a PGG/BHT mixture (400/100 ppm) during the accelerated storage days (30 days) at 65 °C.

IO% is used to monitor the early oxidation stages [30] and to show the anti-peroxidation effect of the PGG extract. All the additives showed the highest inhibitory effect on day 12 of accelerated storage (Figure 3).

As expected, PGG/BHT mix was better than PGG at various concentrations (*p* < 0.05) as it displayed the highest IO% (79.66%), followed by PGG-800 ppm and BHT with IO% of 72.88%. PGG potentiated and significantly enhanced the anti-oxidation effect of BHT on day 12. Although the inhibitory effects of the additives were lower on day 18 of storage, they remained steady till day 24. The oxidation inhibitory ability of the extract was concentration-dependent till day 24. However, it fluctuated on day 30, possibly due to the instability of the antioxidants in the extracts, particularly at low concentrations. In the PGG/BHT mix, PGG-400 ppm stabilized the BHT (100 ppm) till the end of the storage period. The IO% exhibited by PGG-800 ppm and PGG/BHT (46.06%) was similar to that obtained by BHT (56.18%) after 30 days of accelerated storage.

### 3.7. p-Anisidine Value (p-AV)

**Figure 4:**
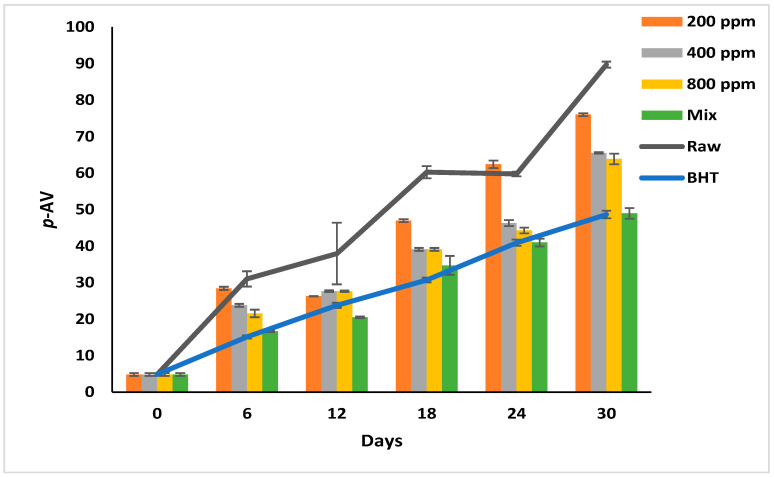
*P*-Anisidine value (*p*-AV) of raw soybean oil (without antioxidants) and soybean oil enriched with PGG leaves extract (200, 400, and 800 ppm), BHT (200 ppm), and a PGG/BHT mixture (400/100 ppm) during the accelerated storage days (30 days) at 65 °C.

*p*-AV reflects the degree of decomposition of the peroxides into short-chain carbonyls, aldehydes, and ketones [6,28]. At this oxidation stage, the oil develops a rancid odor and flavor [6]. The *p*-AV increased progressively in the raw soybean oil during storage from 4.83 ± 0.40 to 89.72 ± 0.83 (Figure 4). Soybean oil produces abundant secondary oxidation products because it is rich in linolenic acid (C18:3) [1].

Although the oils with additives show significantly slower oxidation rates than the raw oil (*p* < 0.05), this effect depends on the additive type and its concentration. The effect of PGG/BHT (400/100 ppm) was similar to that of BHT (*p* > 0.05) in retarding the secondary oxidation product formation, with a *p*-AV of 48.93 and 48.63 on day 30 of storage, respectively. While the PGG extracts inhibited the decomposition of the hydroperoxides in a concentration-dependent manner, PGG-800 ppm showed the lowest *p*-AV (63.87) compared to the other concentrations at the end of the storage period. Consequently, the inhibitory effect of PGG-800 ppm was comparable to that of BHT and PGG/BHT. Our findings are consistent with Phuong et al. [4] and Drinić et al. [28], who observed that rambutan and pomegranate peel extracts (1000 ppm) were as effective as TBHQ in retarding hydroperoxides decomposition. Similarly, the essential oils from *Angelica dahurica cv. Yubaizhi* at 400 ppm [10] and pomegranate peel extract at 500 ppm [28] potentiated the anti-oxidative effect of TBHQ and BHT (100 ppm), respectively.

### 3.8. Total Oxidation Value (TOTOX)

Autooxidation results in various primary and secondary products negatively affecting oil quality. TOTOX reflects the primary and secondary oxidation product content [10] and tracks oxidation progression [6]. By calculating the TOTOX value depending on the peroxide and the para anisidine values, we could theoretically predict the additive’s efficiency in retarding the oxidation process [30].

**Figure 5:**
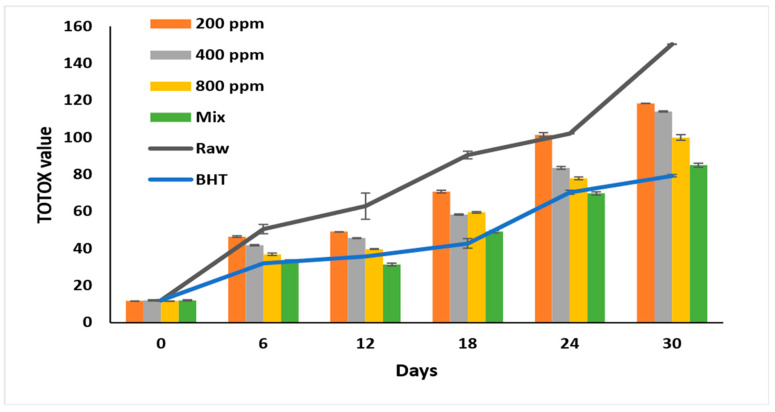
Total oxidation value (TOTOX value) of raw soybean oil (without antioxidants) and soybean oil enriched with PGG leaves extract (200, 400, and 800 ppm), BHT (200 ppm), and a PGG/BHT mixture (400/100 ppm) during the accelerated storage days (30 days) at 65 °C.

The highest oxidation value was recorded for raw oil (*p* < 0.05) at 150.32 ± 0.4 on day 30 of accelerated storage (Figure 5). Adding PGG/BHT (400/100 ppm) retarded the oxidation as effectively as BHT (*p* > 0.05), with total oxidation values of 84.93 ± 0.62 and 79.23 ± 1.05, respectively, at the end of the storage period. PGG extract inhibited soybean oil oxidation in a concentration-dependent manner (*p* < 0.05). PGG-800 ppm retarded the oxidation in soybean oil more efficiently than PGG at 200 ppm and 400 ppm during storage (*p* < 0.05). On day 30 of the storage, PGG-800 ppm inhibited soybean oil oxidation by 33.6% compared to raw oil. Our results agreed with Meng et al. [8], who showed lower TOTOX values of sunflower oil after adding essential oils from *Magnolia liliflora Desr.* (1600 ppm) (195.63), similar to TBHQ-200 ppm (124.31) compared to the raw oil (356.14) after 30 days of accelerated storage—similarly, the mixture of essential oils of *Angelica dahurica cv. Yubaizhi* at 800 ppm and 400 ppm with TBHQ (100 ppm) was as effective as TBHQ (200 ppm) in inhibiting the oxidation of sunflower oil stored for 24 days at 65 °C and reached TOTOX values of 165.8 and 158.5, respectively [10].

### 3.9. Thiobarbituric Acid Reactive Substances (TBARS)

At high temperatures, hydroperoxides react with oxygen to form malondialdehydes (MDA), which impart bad flavor and odor to the oils [6,10]. Therefore, it is used as an oxidative stability indicator [8]. The TBARS test is more sensitive than *p*-AV as it is affected by the presence of ketones, esters, and pyridines [6]. Consequently, this test is used to evaluate the additives’ ability to inhibit the cyclization of cyclic compounds and MDA formation [4].

Like *p*-AV and TOTOX values, PGG/BHT was as effective as BHT (*p >* 0.05) in inhibiting the formation of secondary oxidation products. PGG inhibited MDA formation in a concentration-dependent manner (*p* > 0.05) compared to raw oil.

**Figure 6:**
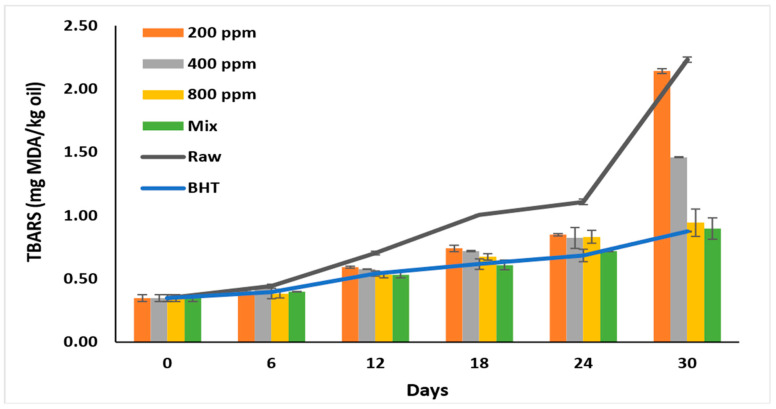
TBARS value (mg MDA/kg oil) of raw soybean oil (without antioxidants) and soybean oil enriched with PGG leaves extract (200, 400, and 800 ppm), BHT (200 ppm), and a PGG/BHT mixture (400/100 ppm) during the accelerated storage days (30 days) at 65 °C.

TBARS recorded the lowest value before storage (0.35 ± 0.03 mg MDA/kg oil) (Figure 6). The formation of secondary products gradually increased till day 6 in all oil samples (*p* > 0.05) and ranged between 0.38–0.44 mg/kg. On day 30, the MDA formation nearly doubled, particularly in the raw oil (2.23 ± 0.00 mg MDA/kg oil) and oil with PGG-200 ppm (2.14 ± 0.02 mg MDA/kg oil). Oil with BHT displayed a TBARS value of 0.87 ± 0.00 mg/kg on day 30. Similarly, PGG/BHT (400/100 ppm) and PGG-800 ppm effectively inhibited MDA formation till day 30 and attained TBARS values of 0.90 ± 0.08 mg/kg, and 0.94 ± 0.11 mg/kg, respectively. The effect of PGG-400 ppm potentiated BHT (100 ppm) in inhibiting the later stages of rancidity. This was consistent with Wang et al. [10] and Drinić et al. [28], who showed that adding essential oils of *Angelica dahurica cv. Yubaizhi* (400 ppm) and pomegranate peel extract (500 ppm) enhanced the effect of TBHQ and BHT, respectively. Similarly, pomegranate peel extract (1000 ppm) [28], essential oils extracted from *Punica granatum cv. Heyinshiliu* peels (800 ppm) [7] and nano-encapsulated olive leaf (300 ppm) [39] significantly inhibited MDA formation in pomegranate, sunflower, and soybean oils, respectively.

### 3.10. Conjugated Dienes (CD) and Conjugated Trienes (CT)

During oxidation, the unsaturated fatty acids’ non-conjugated double and triple bonds are rearranged to form stable conjugated dienes and trienes (CD and CT), respectively [37,40]. High concentrations of the conjugated products cause oxidative deterioration of the oil, indicating the additives’ effectiveness in preventing autoxidation [1]. While CD is the primary oxidation product, CT is a secondary oxidation product that depends on the presence of other secondary oxidation products, aldehydes, and ketones [31,41]. CD and CT levels are used to monitor lipid oxidation [37].

**Figure 7:**
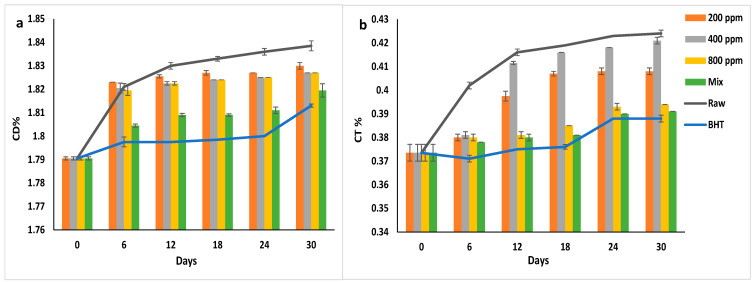
(**a**) Conjugated dienes (CD%) and (**b**) Conjugated trienes (CT%) of raw soybean oil (without antioxidants) and soybean oil enriched with PGG leaf extract (200, 400, and 800 ppm), BHT (200 ppm), and a PGG/BHT mixture (400/100 ppm) during the accelerated storage days (30 days) at 65 °C.

Figure 7a,b illustrate the CD% and CT% of soybean oil enriched with natural and synthetic antioxidants. BHT inhibited the CD formation in soybean oil from day 6 to day 24 of storage (*p* > 0.05) (1.8%) and then increased significantly to 1.81 ± 0.00% on day 30. Similarly, PGG/BHT mix followed BHT in inhibitory efficacy (*p* < 0.05). It inhibited the double bond rearrangement till day 18 (CD = 1.80%). PGG extract at different concentrations slightly reduced the formation of the CD in soybean oil with prolonged storage. However, PGG-800 ppm showed the highest inhibition effect compared to other concentrations (*p* < 0.05).

Similarly, BHT had the highest inhibitory effect on the rearrangement of the triple bonds in the fatty acids (*p* < 0.05). CT% gradually increased from 0.37 ± 0.00% to 0.39 ± 0.00% during the storage. BHT reduced the CT formation by approximately 8.5% compared to the raw oil on day 30 of storage. PGG/BHT and PGG-800 ppm also inhibited CT formation by 7.78% and 7.08%, respectively.

The obtained results were not consistent with the results by El-Hadary and Taha [1], showing that pomegranate peel extract (600 ppm) was more efficient than TBHQ in inhibiting CT and CD formation. However, our results agreed with the study by Wang et al. [10], reporting that the essential oil of *Angelica dahurica cv. Yubaizhi* (800 and 400 ppm) mixed with TBHQ (100 ppm) followed TBHQ (200 ppm) in inhibiting CD and CT formation in sunflower oil stored for 24 days at 65 °C. Moreover, Meng et al. [8] observed that adding 1600 ppm of *Magnolia liliflora Desr.* essential oil significantly inhibited CT and CD formation in sunflower oil compared to the control, albeit not as effectively as TBHQ (200 ppm) after 30 days of accelerated storage.

### 3.11. Refractive Index (RI) ND^20^

**Figure 8:**
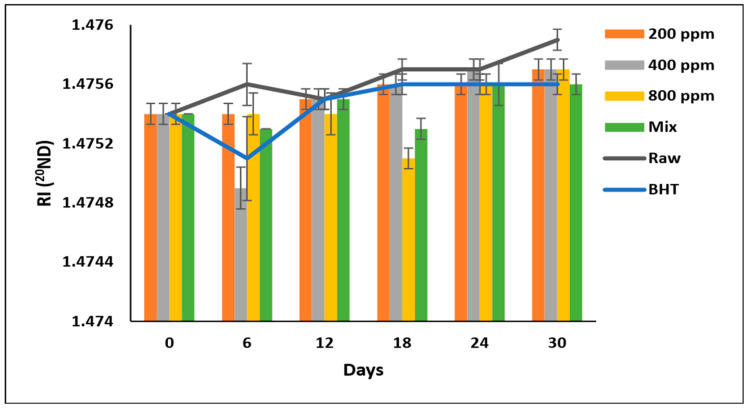
Refractive index (RI) of raw soybean oil (without antioxidants) and soybean oil enriched with PGG leaves extract (200, 400, and 800 ppm), BHT (200 ppm), and a PGG/BHT mixture (400/100 ppm) during the accelerated storage days (30 days) at 65 °C. RI measured at 20 °C and using light with a wavelength of the sodium D line (589.29 nm) and expressed in (^20^ND)

The RI of soybean oil samples fluctuated during the storage period (Figure 8). However, PGG-800 ppm was as effective as BHT and PGG/BHT (*p* > 0.05), with the lowest RI in soybean oil after prolonged storage. Soybean oil enriched with BHT, PGG/BHT, and PGG-800 ppm exhibited RI values of 1.4756, 1.4756, and 1.4757, respectively, compared to 1.4759 of raw soybean oil on day 30. These results disagreed with Neves et al. [5], who reported that the RI of sunflower oil containing *Pterospartum tridentatum* ethanolic extract (500 and 1000 mg/L) was unaffected after 30 days of storage.

### 3.12. Fatty Acids Composition

Oxidation affects the inherent composition of the oils as it converts the polyunsaturated fatty acids (PUSFAs) to monounsaturated and saturated fatty acids (SFAs) and isomerizes the cis to trans fatty acids. By determining the fatty acids composition, we can predict the degree of oxidation [10] and the stability of the oil [8].

As shown in Table 4, raw soybean oil undergoes severe oxidation during storage. After 30 days of accelerated storage at 65 °C, the SFAs of raw soybean oil nearly doubled (35.52%). The oxidation of the raw oil was also indicated by the high oleic/linoleic ratio (4.34) and low linoleic/palmitic ratio (0.17). At a low concentration (200 ppm), PGG weakly inhibited the autooxidation process as the SFAs rapidly increased to 20.11%. However, PGG-800 ppm inhibited the transformation of the PUSFAs to SFAs, as efficiently as BHT compared to the raw oil, indicating its potent antioxidant effect during storage. Moreover, the results confirmed that PGG extract (400 ppm) was synergic with BHT (100 ppm) as PGG/BHT mix effectively suppressed the increase in oleic acid and saturated fatty acid content compared to BHT only. Our results agreed with Wang et al. [10], showing that the essential oil of Angelica dahurica cv. Yubaizhi (800 ppm and 400 ppm) mixed with TBHQ (100 ppm) had the same effect as TBHQ (200 ppm) in inhibiting the transformation of oleic and linoleic acids into saturated acids [10]. Similarly, the essential oils extracted from *Punica granatum cv. Heyinshiliu* peels (800 ppm) [7] and *Magnolia liliflora Desr.* (1600 ppm) [8] suppressed the conversion of the USFAs to SFAs.

## 4. Conclusions

Although previous research proved that agro-waste has robust antioxidant activity, plant by-products, mainly leaves, are underutilized. Besides, finding a sustainable natural antioxidant source in the oil industry becomes vital due to consumer awareness about the adverse effects of synthetic antioxidants. In this context, we investigated the potential antioxidant activity of a mixture of the lyophilized Pomegranate-Guava-Grape leaves water extract (1:1:1) (PGG) to be utilized as a suitable alternative to the BHT in the oil industry.

The mixture of lyophilized pomegranate-guava-grape leaves water extract (1:1:1) (PGG) exhibited high phenolic and flavonoid content, with ellagic acid and naringenin being the most abundant phenolic acid and flavonoid, respectively. Moreover, the extract mixture displayed a synergistic scavenging effect toward DPPH^•^. Therefore, PGG extract could be potentially used as a sustainable and effective natural antioxidant source.

The concentration-dependent effect of PGG in retarding the rancidity of soybean oil during storage was comparable to that of synthetic BHT. PGG at 800 ppm enhanced the oxidative stability of soybean oil compared to raw oil. The extract also effectively reduced lipids’ hydrolysis, polymerization, and oxidation. Soybean oil enriched with PGG-800 ppm possessed low acid, peroxide, *p*-anisidine, TBARS, and conjugated product values. Interestingly, when combined with PGG extract, the BHT amount used in the oil industry can be reduced as PGG-400 ppm acts synergistically with BHT (100 ppm). PGG/BHT (400/100 ppm) retarded the autooxidation process during accelerated storage. Therefore, we believe that using PGG extract at a concentration of 800 ppm or higher would be more efficient in extending the shelf-life of soybean oil during storage.

## Figures and Tables

**Table 1:** Soybean oil samples’ preparation.

Oil Samples	PGG Extract %	BHT %
Raw oil	0	0
200 ppm oil	0.02	0
400 ppm oil	0.04	0
800 ppm oil	0.08	0
BHT oil	0	0.02
Mix oil	0.04	0.01

ppm (part per million); BHT (butylated hydroxytoluene); PGG% (a mixture of lyophilized pomegranate leaves, guava leaves, and grape leaves extracts (1:1:1)).

**Table 2:** Bioactive components and antioxidant activity of pomegranate leaves, guava leaves, grape leaves, and pomegranate-guava-grape (PGG) mixture leaves (1:1:1) extracts.

Extracts	Pomegranate Leave	Guava Leaves	Grape Leave	PGG Mixture
Total phenolics (mg/g)	248.51 ± 90.45 ^a^	44.99 ± 18.62 ^b,c^	31.61 ± 0.49 ^c^	122.95 ± 4.42 ^b^
Total flavonoids (mg/g)	94.12 ± 0.91 ^a^	51.45 ± 0.82 ^c^	46.60 ± 0.91 ^d^	60.10 ± 0.27 ^b^
DPPH^•^ Ic_50_ (µg/mL)	11.11 ± 0.02 ^c^	42.61 ± 0.27 ^b^	73.92 ± 0.93 ^a^	8.19 ± 0.08 ^d^
ABTS^•^ Ic_50_ (µg/mL)	0.58 ± 0.02 ^d^	7.19 ± 0.04 ^b^	7.67 ± 0.04 ^a^	1.78 ± 0.08 ^c^

Data are presented in mean ± standard deviation (SD); Mean values in a row having different superscripts (a–d) are significantly different at (*p* < 0.05).

**Table 3:** Phenolic profile of pomegranate-guava-grape leaves (PGG) extract.

Phenolic Acids and Phenolic Derivatives (mg/g)	
Ellagic acid	27.38
Chlorogenic acid	10.30
Gallic acid	4.03
Caffeic acid	1.72
Pyro catechol	0.74
Ferulic acid	0.42
Cinnamic acid	0.01
Vanillin	0.01
**Flavonoids (mg/g)**	
Naringenin	0.95
Rutin	0.49
Quercetin	0.22
Daidzein	0.06

**Table 4:** Fatty acids composition (%) of soybean enriched with PGG extract, BHT, and PGG/BHT during accelerated storage for 30 days at 65 °C.

Ample	Days	C_16_:0	C_17_:0	C_18_:0	C_18_:1	C_18_:2	C_20_:0	C_18_:3 (γ)	C_20_:1	C_18_:3 (α)	C_22_:0	SFAs	UFAs	C_18_:1/C_18_:2	C_18_:2/C_16_:0
Raw oil	0	11.92	-	4.33	25.08	50.37	-	1.79	1.46	5.02	0.03	16.28	83.72	0.09	4.23
	6	12.28	-	3.99	24.42	50.90	-	1.62	1.73	5.06	-	16.27	83.73	0.08	4.15
	30	17.34	1.18	12.69	35.76	2.93	4.31	4.76	10.81	10.23	-	35.52	64.48	4.34	0.17
200 ppm	0	11.92	-	4.33	25.08	50.37	-	1.79	1.46	5.02	0.03	16.28	83.72	0.09	4.23
	6	12.20	-	4.01	24.92	50.74	0.41	1.42	1.46	4.83		16.62	83.38	0.08	4.16
	30	12.39	3.29	4.43	25.44	48.67	-	1.34	0.00	4.45	-	20.11	79.89	0.09	3.93
400 ppm	0	11.92	-	4.33	25.08	50.37	-	1.79	1.46	5.02	0.03	16.28	83.72	0.09	4.23
	6	12.48	-	4.41	24.66	50.80	-	1.24	1.32	5.09	-	16.89	83.11	0.09	4.07
	30	11.72	2.90	3.84	23.95	49.26	-	1.44	1.72	5.16	-	18.47	81.53	0.08	4.20
800 ppm	0	11.92	-	4.33	25.08	50.37	-	1.79	1.46	5.02	0.03	16.28	83.72	0.09	4.23
	6	11.81	2.59	4.13	24.11	49.53	-	1.21	1.40	5.23	-	16.89	83.11	0.08	4.20
	30	12.50	-	3.96	26.12	49.48	-	1.21	1.17	4.78	0.79	17.25	82.75	0.08	3.96
BHT	0	11.92	-	4.33	25.08	50.37	-	1.79	1.46	5.02	0.03	16.28	83.72	0.09	4.23
	6	12.18	-	4.03	25.08	51.24	-	1.13	1.40	4.94	-	16.22	83.78	0.08	4.21
	30	11.41	3.08	4.01	24.04	49.42	0.88	1.19	1.23	4.75	-	19.37	80.63	0.08	4.33
Mix	0	11.92	-	4.33	25.08	50.37	-	1.79	1.46	5.02	0.03	16.28	83.72	0.09	4.23
	6	12.18	-	4.03	25.08	51.24	-	1.13	1.40	4.94	-	16.22	83.78	0.08	4.21
	30	12.17	-	3.82	22.32	54.94	-	1.21	1.08	4.46		16.49	83.51	0.07	4.51

(-) Not detected; (C_16_:0) Palmitic acid; (C_17_:0) Heptadecanoic acid; (C_18_:0) Stearic acid; (C_18_:1) Oleic acid; (C_18_:2) Linoleic acid; (C_20_:0) Arachidic acid; (C_18_:3 (γ)) Gamma linolenic acid; (C_20_:1) Eicosenoic acid; (C_18_:3 (α)) Alpha linolenic acid; (C_22_:0) Behenic acid. (SFAs) Saturated fatty acids; (USFAs) unsaturated fatty acids. C_18_:1/C_18_:2 is the ratio between oleic acid and linoleic acid; C_18_:2/C_16_:0 is the ratio between linoleic acid and palmitic acid.

## Data Availability

The data are contained within the article.
